# Maternal dietary DHA and EPA supplementation ameliorates adverse cardiac outcomes in THC-exposed rat offspring

**DOI:** 10.1038/s41598-025-92844-6

**Published:** 2025-03-10

**Authors:** Kendrick Lee, Mohammed H. Sarikahya, Samantha L. Cousineau, Ken K.-C. Yeung, Amica Lucas, Kara Loudon, Thane Tomy, Gregg T. Tomy, David R. C. Natale, Steven R. Laviolette, Daniel B. Hardy

**Affiliations:** 1 Department of Physiology and Pharmacology, Western University, London, Canada; 2 Department of Obstetrics and Gynecology, Schulich School of Medicine and Dentistry, London, Canada; 3 Children’s Health Research Institute, London, Canada; 4 Department of Anatomy and Cell Biology, Schulich School of Medicine and DentistryWestern University, London, Canada; 5 Department of Chemistry and Biochemistry, Western University, London, Canada; 6 University of Manitoba, Winnipeg, MB Canada; 7 Queen’s University, Kingston, ON Canada; 8 Department of Physiology and Pharmacology, University of Western Ontario, Dental Sciences Building Room 2023, London, ON N6A 5C1 Canada

**Keywords:** Cannabis, Maternal, Omega-3 fatty acids, Cardiac, Δ9-tetrahydrocannabinol (THC), Endocannabinoid system, Disease model, Intrauterine growth, Cardiovascular biology, Metabolism, Reproductive biology

## Abstract

Cannabis use in pregnancy is associated with low birthweight outcomes. Recent preclinical data suggests that maternal Δ9-tetrahydrocannabinol (THC) exposure leads to decreases in birthweight followed by early cardiac deficits in offspring. Currently, no studies have explored an intervention for these maternal THC-induced deficits. Omega-3 fatty acids have been shown to exhibit cardioprotective effects. In this present study, we demonstrated that maternal dietary supplementation of omega-3 fatty acids ameliorates both THC-induced fetal growth and postnatal cardiac deficits in offspring. Our data indicates this may be underpinned by alterations in cardiac and hepatic fatty acids and reduction in markers of cardiac collagen deposition. Interestingly, the cardioprotective effects of omega-3s may be further underscored by decreased signaling of the cardiac endocannabinoid system. With increasing rates of cannabis use in pregnancy and recent evidence of subsequent cardiometabolic aberrations in offspring, our data suggests a potential intervention for THC-induced fetal growth and cardiac disturbances in offspring.

**Supplementary Information:**

The online version contains supplementary material available at 10.1038/s41598-025-92844-6.

## Introduction

The use of Cannabis during pregnancy is increasing with an estimated prevalence of 3.4% in 2012 and 7% in 2017, in the US^1^. Numerous studies suggest that self-reported prevalence of cannabis consumption in pregnancy can range anywhere between 2 and 35% depending on the population demographics, definition of use, and method of detection for cannabis^1–6^. Clinical reports indicate that the reason for cannabis use in pregnancy could be due to symptom management of chronic conditions (i.e. pain and mental health) and pregnancy-related nausea and vomiting^7^. Furthermore, the uncertainty surrounding the negative effects of cannabis on postnatal outcomes may be a major driver for continued use during pregnancy^8^. This is of great concern given clinical studies have demonstrated that prenatal cannabis use may lead to smaller and/or low birthweight babies^9–14^. According to the fetal programing hypothesis, these offspring may be at an increased risk for cardiovascular disease later in life^15–18^.

THC is the major psychoactive constituent in cannabis and can readily cross the placenta. It has been previously demonstrated that exposure to 3 mg/kg/day THC in rat dams leads to fetal growth restriction (FGR) and complete catch-up growth by three-weeks of age^19^. While catch-up growth may appear to be beneficial for the neonate, one of the earliest epidemiological studies linking birth weight to cardiovascular disease indicates it further exacerbates cardiovascular risk^16^. In a follow-up study, we demonstrated that THC-exposed animals that caught-up in growth also exhibited modest increases in ventricular wall thickness and increases in markers of cardiac remodelling concomitant with a reduction in cardiac function^20^. These functional deficits at three weeks of age were supported by a subsequent study in mouse offspring^21^. However, to date, intervention strategies to reduce THC-induced fetal growth and cardiac deficits in the offspring remain elusive.

As a feasible and potentially efficacious intervention, there is considerable evidence to support the cardioprotective effects of omega-3 fatty acids. For instance, meta-analysis has demonstrated that omega-3 long-chain poly unsaturated fatty acids (PUFAs), docosahexaenoic acid (DHA) and eicosapentaenoic acid (EPA), may reduce cardiovascular mortality and improve cardiovascular function^22^. With respect to cardiac remodelling, several clinical studies have shown promise for heart failure hospitalization, cardiac fibrosis and improved cardiac function^23–26^. Likewise, preclinical studies have demonstrated that DHA and/or EPA can reduce cardiac hypertrophy, fibrosis and ultimately improve cardiac function^27–31^. Based on these preclinical studies, improving cardiac fatty acid composition, and hampering the inflammatory response may be key to modulating adverse cardiac remodelling with DHA and EPA. Moreover, it is conceivable that supplementation with maternal omega fatty acids could further ameliorate THC-induced cardiac deficits by preventing fetal growth deficits in itself. Although the data is mixed, clinical studies indicate that omega-3s may be able to increase fetal growth trajectories and fetal weights^32,33^. Similarly, maternal omega-3 supplementation has been shown to increase fetal weights in rodents^34^, while another study found that it ameliorated LPS-induced FGR^35^. Interestingly, clinical studies have demonstrated that, in growth-restricted infants/children, exposure to omega-3s early in life can mitigate cardiovascular risk^36–38^.

THC is a known partial agonist to the receptors in the canonical endocannabinoid system. This system is present in peripheral tissue such as the heart and is comprised of cannabinoid receptors (type 1 (CB1) and type 2 (CB2)), endocannabinoid ligands and their respective synthesizing and degrading enzymes. Depending on the disease model, an active endocannabinoid system is thought to be associated with the pathophysiology in various models of cardiovascular disease (as reviewed in ref)^39–42^. We have previously demonstrated that prenatal exposure to another major phytocannabinoid, cannabidiol, can lead to reduced cardiac function concomitant with a decrease in cardioprotective CB2^43^. In addition, there is emerging evidence to suggest that the cardioprotective mechanisms of DHA and EPA may involve the endocannabinoid system^44^. It is speculated this may be due to alternations in AA-derived endocannabinoids by DHA and EPA, in addition to its ability to form endocannabinoid conjugates that preferentially act on CB2^44^. Given the potential role for DHA and EPA to benefit fetal growth and cardiovascular health, we hypothesize that the fetal growth deficits and postnatal cardiac deficits can be ameliorated by maternal dietary supplementation of DHA and EPA. Further, we postulate this may benefit the heart via alterations in the cardiac endocannabinoid system.

## Results

### Gestational exposure to THC with or without DHA and EPA dietary supplementation does not impact overall maternal outcome measurements

Pregnant rats were administered 3 mg/kg i.p. of THC daily from gestational day 6–22, with or without the DHA + EPA-enriched diet. This diet was given from time of arrival at the animal facility (GD3) until the pups were weaned at PND21. To assess maternal outcomes, gestational length, food intake, weight gain and litter size at birth were measured, as previously published^19,43^. There were no significant differences in these maternal outcome measurements between the 4 groups; however, with respect to maternal food intake, there was a significant drug effect at the earlier timepoint (GD 12–14, *p* = 0.0339; Table 1).

**Table 1:** Maternal outcome measurements.

Maternal Measurements	Control Diet		DHA + EPA Diet		Significant *P* Values (Drug/diet effects + interactions)
	VEH (n = 8)	THC (n = 8)	VEH (n = 8)	THC (n = 8)	
Gestation Length (days)	22	22	22	22	
Average Food Intake: GD12-14 (g/day)	21.8 ± 3.7	20.2 ± 3.8	20.9 ± 1.3	17.9 ± 1.8	P_T_<0.05
Average Food Intake: GD18-20 (g/day)	22.3 ± 1.6	21.8 ± 3.1	24.3 ± 1.9	21.6 ± 2.1	
Average Weight Gain: GD12-14 (g/day)	12.8 ± 4.4	12.1 ± 2.7	14.0 ± 3.2	15.4 ± 6.0	
Average Weight Gain: GD18-20 (g/day)	24.5 ± 5.7	25.9 ± 5.2	29.8 ± 3.3	25.13 ± 3.4	
Litter size at Birth	11.6 ± 2.0	11.3 ± 1.6	12.4 ± 1.3	10.6 ± 1.2	

Data represented as means ± SD.

Statistical significance was assessed using a 2-way ANOVA with post-hoc Tukey’s.

P_T_<0.05, Drug Effect (VEH vs. THC).

### The effects of gestational THC with or without DHA and EPA dietary supplementation on fetal and postnatal growth

**Fig. 1:**
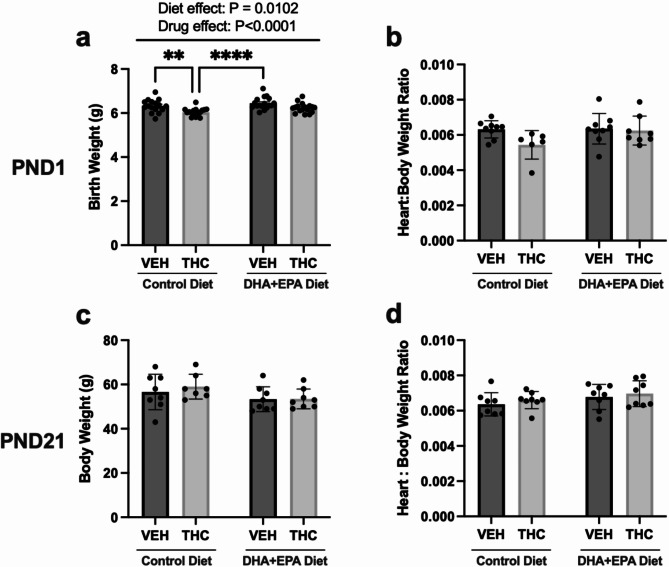
Maternal DHA + EPA-enriched diets ameliorate fetal growth deficits in THC-exposed offspring. (**a**) Postnatal day 1 (PND1) body weight, (**b**) PND1 heart to body weight ratio, (**c**) Postnatal day 21 (PND21) body weight, and (**d**) PND21 heart to body weight ratio. Each PND1 body weight data point represents an average of 3–4 pups from 1 dam, thus data is represented as means ± SEM. All other data represented as means ± SD, *n* = 6–9/group for PND1 heart: body weight, *n* = 7–8/group for all PND21 weight analysis. For graph (**b**), (**c**), and (**d**), each data point represents an offspring from a single and different dam. Significance was determined by a two-way ANOVA post-hoc Tukey’s, **P < 0.01,****P < 0.0001. Significant diet effect (control diet vs. DHA + EPA diet), drug effect (VEH vs. THC) and interaction are labelled at the top of the respective graphs, where applicable.

To examine if THC and/or our DHA and EPA-enriched diet impacts fetal growth, we measured body weight and heart to body weight ratios at birth. At birth, maternal THC exposure leads to a significant reduction in body weights relative to both vehicle groups (*p*=0.0073 for control diet:VEH vs. control diet:THC and *p*<0.0001 for control diet:THC vs. DH*A+EPA*  diet:VEH) irrespective of diet (Fig. 1a). Interestingly, these deficits were not observed in animals exposed to THC with the DHA + EPA-supplemented diet. There was also a diet dependent increase in birthweight in offspring exposed to the DHA + EPA diet (*p* = 0.0102). The heart to body weight ratios were not significantly changed in offspring exposed to THC alone relative to vehicle alone (Fig. 1b). By three weeks of age, THC-exposed offspring caught-up in growth (Fig. 1c) demonstrated by similar body weights to the vehicle control group. Due to improved fetal growth, offspring exposed to both THC and the diet did not exhibit “catch-up” growth at PND21 (Fig. 1c). No changes were observed in heart to bodyweight ratios at PND21 between groups (Fig. 1d).

### Maternal DHA and EPA diet supplementation ameliorates reductions in cardiac function observed in PND21 male offspring exposed to THC in gestation

To assess cardiac function, echocardiography was employed on vehicle and THC-exposed PND21 male offspring with and without DHA + EPA dietary supplementation. Similar to our previous study^20^, gestational THC exposure leads to a significant reduction in stroke volume (*p* = 0.0037), cardiac output (*p* = 0.0045), and ejection fraction (*p* = 0.0021) relative to the vehicle group in PND21 male offspring (Table 2). Intriguingly, these deficits were not observed in offspring exposed to both THC and the DHA + EPA diet (Table 2). Notably, there were marked improvements in parameters such as stroke volume and cardiac output in offspring exposed to the maternal DHA + EPA diet relative to offspring exposed to THC with control diet (Table 2). In addition, there was a significant diet effect on stroke volume (*p* = 0.0016) and cardiac output (*p* = 0.0012). There was also a significant interaction in contractile function (i.e. EF *p* = 0.031; Table 2). Furthermore, a significant diet effect on LV mass was observed in the DHA + EPA-exposed offspring relative to control diet groups (Table 2).

**Table 2:** Echocardiography data in PND21 offspring.

	Control Diet		DHA + EPA Diet		Significant *P* Values (Drug/diet effects + interactions)
	VEH (n = 11)	THC (*n* = 11)	VEH (*n* = 7)	THC (*n* = 7)	
Heart Rate (BPM)	397.3±50.3	370.8±33.9	360.5±36.6	356.9±40.9	n/a
Stroke Volume (µL)	73.0±2.6	58.9±9.7**^$$$$†^	81.2±15.2	71.5±4.5	P_T_=0.0004, P_D_=0.0016
Cardiac Output (µL/beat)	29.0±3.7	22.8±4.2**^$^	28.9±4.0	25.6±3.7	P_D_=0.0012
Ejection Fraction (%)	82.1±6.4	67.7±12.1**	75.9±9.1	75.0±5.7	P_T_=0.016, P_TD_=0.031
Fractional Shortening (%)	50.6±11.9	43.7±10.8	41.2±8.7	45.1±8.4	n/a
LVAWd (mm)	1.18±0.18	1.17±0.12	1.36±0.088	1.17±0.21	n/a
LVAWs (mm)	2.02±0.28	1.91±0.26	2.08±0.18	1.94±0.18	n/a
LVIDd (mm)	4.16±0.34	4.31±0.35	4.38±0.57	4.42±0.37	n/a
LVIDs (mm)	2.04±0.51	2.44±0.50	2.60±0.67	2.44±0.50	n/a
LVPWd (mm)	1.10±0.19	1.17±0.17	1.28±0.26	1.18±0.15	n/a
LVPWs (mm)	1.86±0.21	1.86±0.39	1.87±0.30	1.89±0.23	n/a
LV vol d (µL)	76.9±14.9	83.2±16.3	88.7±28.5	89.2±17.8	n/a
LV vol s (µL)	15.2±9.5	22.0±11.0	27.1±18.5	22.3±11.3	n/a
LV mass (g)	205.4±48.8^$^	227.9±50.0	277.7±46.7	236.4±41.2	P_D_=0.018

Data represented at means ± SD.

Statistical significance was assessed using a 2-way ANOVA with post-hoc Tukey’s.

*P < 0.05, **P < 0.01, vs. respective VEH group.

^$^*P* < 0.05, ^$$$$^*P* < 0.0001 vs. opposite diet VEH group.

^†^*P* < 0.05, vs. opposite diet THC group.

P_T_, VEH vs. THC animal (Drug Effect).

P_D_, Control diet vs. DHA + EPA diet (Diet Effect).

P_TD_, Interaction.

n/a, not applicable.

LV, left ventricle; LVAWs, left ventricular anterior wall systole; LVAWd, left ventricular.

anterior wall diastole; LVIDs, left ventricular internal diameter systole; LVIDd, left ventricular.

Internal diameter diastole; LVPWs, left ventricular posterior wall systole; LVPWd, left ventricular.

posterior wall diastole; LV vol d, left ventricular volume diastole; LV vol s, left ventricular.

volume systole.

### Maternal DHA and EPA-enriched diet boosts hepatic and cardiac DHA and/or EPA while decreasing arachidonic acid in male PND21 vehicle and THC-exposed rat offspring

**Fig. 2:**
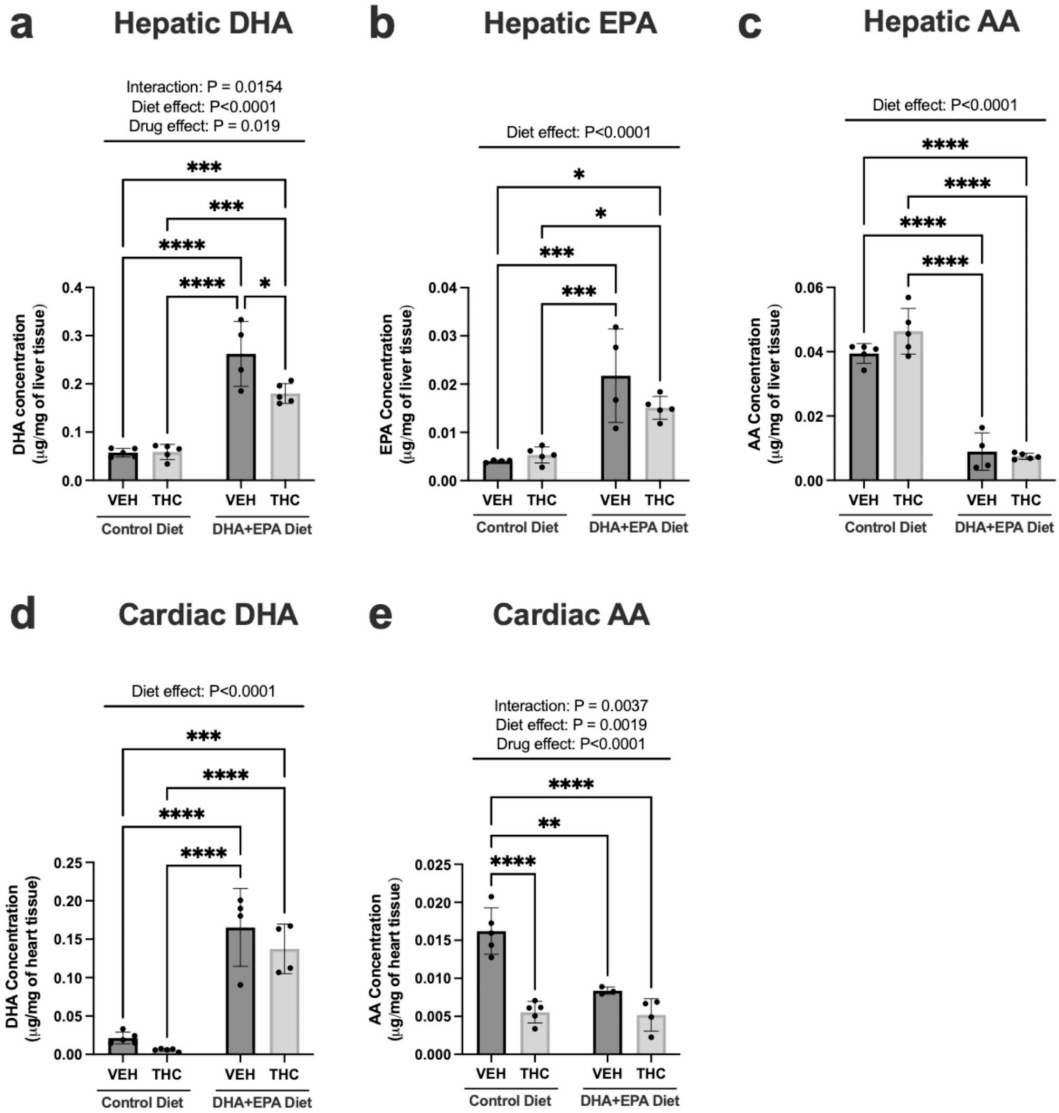
Maternal DHA + EPA diet alters key hepatic and left ventricular omega-6 and omega-3 fatty acids in PND21 vehicle and THC-exposed offspring. Upon MS/MS, EPA could not be accurately detected in the hearts. Concentration of hepatic (**a**) DHA, (**b**) EPA and (**c**) AA. Concentration of cardiac (**d**) DHA and (**e**) AA. Data are represented as means ± SD, *n* = 4–5 males/treatment. Each data point represents a male offspring heart/liver from a single and different dam. Significance was determined by two-way ANOVA followed by Tukey’s post-hoc. *P < 0.05, **P < 0.01, ***P < 0.001, ****P < 0.0001. Significant diet effect (control diet vs. DHA + EPA diet), drug effect (VEH vs. THC) and interaction are labelled at the top of respective graphs, where applicable.

DHA and EPA are well-known long-chain PUFAs that have demonstrated promising cardioprotective potential, in part due to its anti-inflammatory effects^27–31^. In contrast, higher cardiac arachidonic acid (AA) has been implicated in models of heart failure^27^ and underlie increases in pro-inflammatory lipid mediators (i.e., prostaglandins). Given this, we sought to determine whether these beneficial omega-3s were accumulating in offspring livers and hearts. We demonstrated that the DHA + EPA diet led to significantly higher hepatic DHA (*p* < 0.0001) and EPA (*p* < 0.0001) (Fig. 2a & b). Meanwhile, the effects were opposite with AA, with significant reductions in the DHA + EPA diet groups relative to groups exposed to the control diet (*p* < 0.0001) (Fig. 2c). Next, we examined whether these hepatic DHA and EPA increases also translated into the heart. Indeed, there were also similar effects in cardiac DHA with VEH and THC groups supplemented with DHA + EPA diet as they demonstrated substantial increases in cardiac DHA (diet effect, *p* < 0.0001; Fig. 2d). Unfortunately, EPA concentrations were low in the hearts and upon MS/MS, we could not accurately indicate the presence of EPA. As for cardiac AA, there were marked decreases in AA in the DHA + EPA-supplemented groups. Interestingly, the THC group in the control diet also demonstrated significant decreases in AA relative to offspring exposed to the vehicle with control diet (*p* < 0.0001; Fig. 2e).

### Maternal THC exposure leads to increases in transcript abundance of Tnfα while an omega-3-enriched diet induces a diet dependant decrease in Ccl-2

**Fig. 3:**
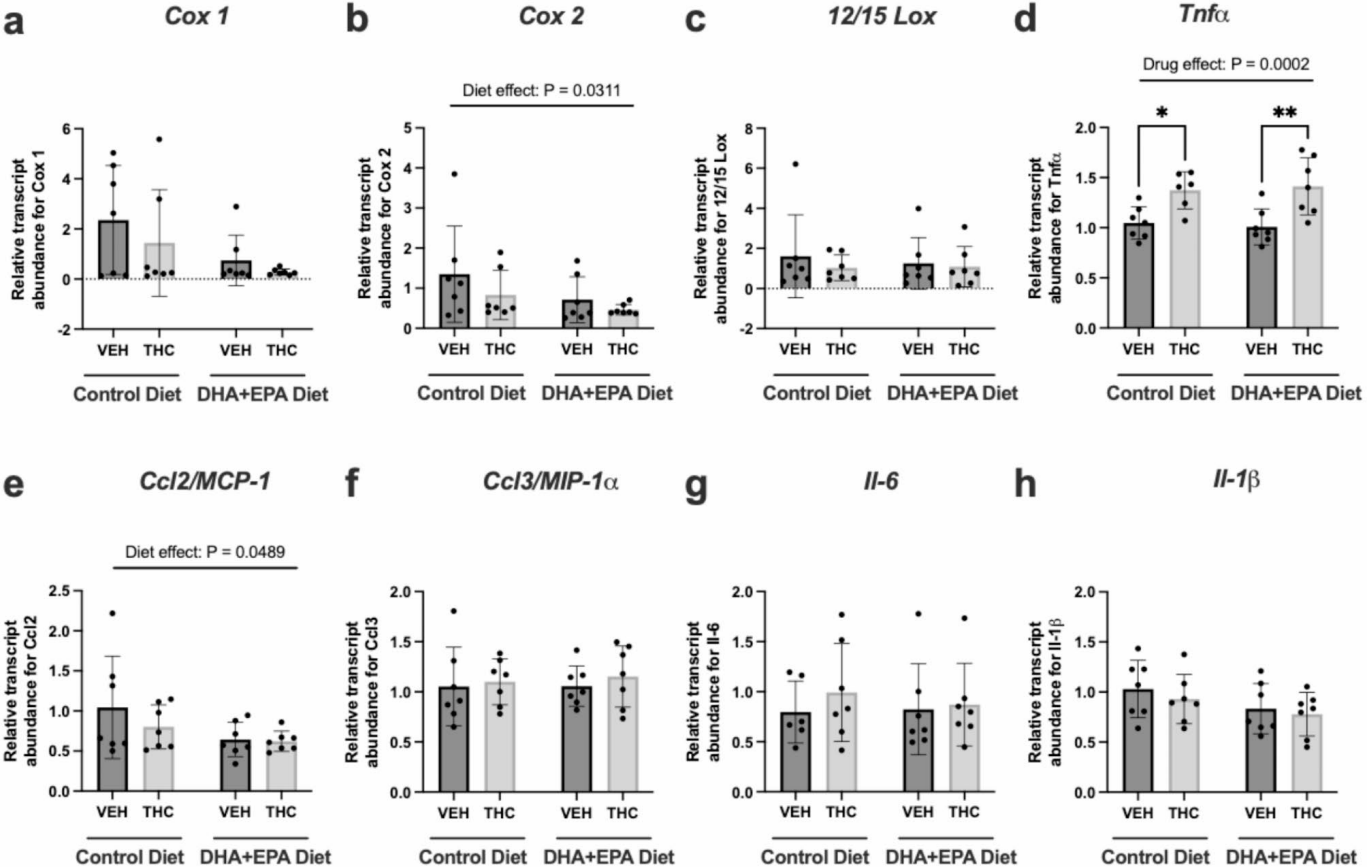
Maternal THC and DHA + EPA-enriched diet differentially alters transcript abundance of cytokines in PND21 vehicle and THC-exposed offspring. RT-qPCR was used to measure relative transcript abundance of major enzymes that generate lipid mediators namely, (**a**) Cox 1, (**b**) Cox 2 and (**c**) 12/15 Lox, as well as major inflammatory cytokines such as, (**d**) Tnfα, (**e**) Ccl2, (**f**) Ccl3, (**g**) Il-6 and (**h**) Il-1β. All targets were normalized to both *β-actin* and *Gapdh*. Data is represented as means mean ± SD, *n* = 7/treatment group. Each data point represents a male offspring heart from a single and different dam. Significance was determined by two-way ANOVA post-hoc Tukey’s. *P < 0.05, **P < 0.01. Significant diet effect (control diet vs. DHA + EPA diet), drug effect (VEH vs. THC) and interaction are labelled at the top of respective graphs, where applicable.

The LOX and COX pathways are well-established to generate lipid mediators from DHA, EPA and AA which have various roles in an inflammatory response. Thus, we measured the mRNA levels of the major enzymes (i.e. Lox and Cox) in heart tissue. Cox2 is an enzyme produces inflammatory lipids from AA which can initiate inflammation and leukocyte infiltration. With respect to post-hoc group comparisons, we observed no changes in the mRNA levels of Cox2 mRNA, however, a significant diet-dependant decrease (*p* = 0.0311) was observed (Fig. 3b). Cox1, the constitutively expressed cyclooxygenase involved in physiological prostaglandin production, was unaltered in male offspring hearts (Fig. 3a). Another target is the 12/15 LOX pathway which produces different DHA-derived mediators (i.e. resolvins and protectins) and AA-derived pro-inflammatory lipid mediators (i.e. 12-HETE and leukotrienes) with various roles in inflammation. No significant changes in mRNA expression of 12/15 LOX were observed across all groups (Fig. 3c).

Next, we followed up with cardiac mRNA levels of cytokines to examine whether there was evidence of a pro-inflammatory response. Offspring exposed to maternal THC alone demonstrated significant increases in mRNA expression of cardiac Tnfα relative to VEH control (*p* = 0.0469), however this was not ameliorated in offspring exposed to THC with the DHA + EPA diet (Fig. 3d). Notably, a significant diet-dependant decrease in cardiac Ccl2 (MCP-1) mRNA (*p* = 0.0489) was observed in offspring exposed to the maternal DHA + EPA-enriched diet (Fig. 3e). The mRNA expression of other inflammatory cytokines such as Ccl3 (Fig. 3f), Il-6 (Fig. 3g) and, Il-1β (Fig. 3h) exhibited no significant differences between groups.

### Gestational THC-induced increases in cardiac collagen content in three-week-old offspring hearts are ameliorated with a maternal diet supplemented with DHA and EPA

**Fig. 4:**
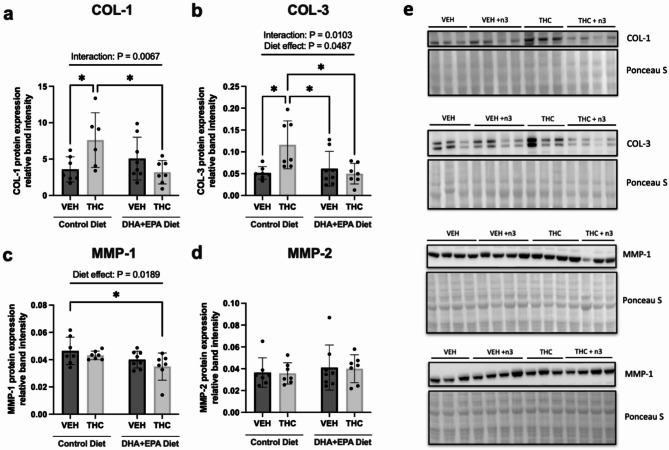
Maternal DHA + EPA significantly reduced collagen content in offspring hearts exposed to THC in gestation. Relative protein expression of markers of cardiac extracellular matrix remodelling, (**a**) COL-1, (**b**) COL-3, (**c**) MMP1 and (**d**) MMP-2. (**e**) Representative immunoblots for respective targets. All protein data was normalized to Ponceau S total protein stain. Each immunoblot target shown is the representative from one out of two immunoblots performed. A pooled sample was loaded into the last lane on the right (cropped out) to normalize relative band intensity between the two blots. Full-length blots can be found in **Supplementary Fig. ****S1****-S4**. Data are represented at means ± SD, *n* = 7–8 males hearts/treatment group. Each data point represents a male offspring heart from a single and different dam. Significance was determined by a two-way ANOVA post-hoc Tukey’s, *P < 0.05, **P < 0.01. Significant diet effect (control diet vs. DHA + EPA diet), drug effect (VEH vs. THC) and interaction are labelled at the top of respective graphs, where applicable.

Given inflammation is known to orchestrate cardiac remodeling (e.g. extracellular matrix remodeling), we next sought to determine whether this was involved in the THC-induced deficits in cardiac function, as previously demonstrated^20^. In particular, we examined whether prenatal THC exposure, leads to increases in collagen content (as reported previously^20^). In PND21 offspring, the protein levels of collagen type 1 (COL-1) and type 3 (COL-3), in VEH and THC LV tissue, were differentially altered when exposed to our intervention diet (significant interaction *p* = 0.0067 and *p* = 0.0103, respectively; Fig. 4a **& b**). Specifically, offspring exposed to THC during gestation had significantly higher protein levels of COL-1 (*p* = 0.0496) and COL-3 (*p* = 0.0152) relative to the vehicle control diet group (Fig. 4a **& b**). When animals were given THC with our DHA + EPA diet, we observed significant reductions in protein expression of COL-1 and COL-3 relative to offspring exposed to THC with the control diet (*p* = 0.0268 and *p* = 0.0122, respectively; Fig. 4a **& b**). We next examined whether matrix metalloproteinases (MMPs) were altered in three-week offspring. After a cardiac insult, MMPs can contribute to processes including initial injury and repair, the onset and resolution of inflammation, the activation and de-activation of myofibroblasts, and the deposition and breakdown of ECM. Associated with the changes in collagen, the DHA + EPA diet led to a significant diet-effect decrease in MMP-1 (*p* = 0.0189), specifically between vehicle with control diet versus THC with DHA + EPA diet (Fig. 4c). No changes were observed for MMP-2 (Fig. 4d). All representative blots are found in Fig. 4e.

### Maternal omega-3 diet alters the cardiac endocannabinoid system in PND21 vehicle and THC-exposed offspring 

**Fig. 5:**
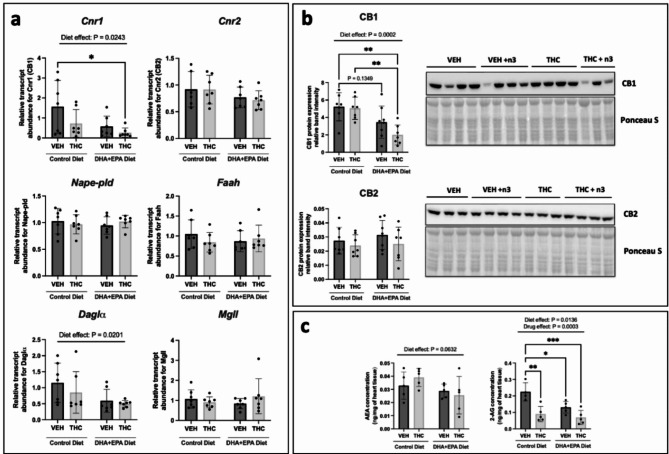
Maternal DHA + EPA-enriched diets alter the transcript, protein and endocannabinoid levels of the canonical endocannabinoid system in PND21 vehicle and THC-exposed offspring hearts. (**a**) Transcript abundance of cannabinoid receptors and enzymes responsible for endocannabinoid turnover. All transcript data is normalized to two housekeeping genes, *β-actin* and *Gapdh*. (**b**) Protein expression with represented immunoblots of cannabinoid receptors CB1 and CB2. All protein data was normalized to Ponceau S total protein stain. Each immunoblot target shown is the representative from one out of two immunoblots performed. A pooled sample was loaded into the last lane on the right (cropped out) to normalize relative band intensity between the two blots. Full-length blots can be found in **Supplementary Fig. S5 & S4**. (**c**) HPLC quantification of endocannabinoids, AEA (left) and 2-AG (right) relative to tissue weight. Data are represented at means ± SD. For panels (**a**) and (**b**) *n* = 7–8 males hearts/treatment group. For panels (**c**) *n* = 5 males hearts/treatment group. Each data point represents a male offspring heart from a single and different dam, Significance was determined by a two-way ANOVA post-hoc Tukey’s, *P < 0.05, **P < 0.01, ***P < 0.001. Significant diet effect (control diet vs. DHA + EPA diet), drug effect (VEH vs. THC) and interaction are labelled at the top of respective graphs, where applicable.

Given that THC and dietary DHA + EPA can influence the ECS^44^, we next measured the transcript levels of the canonical endocannabinoid system in three-week heart tissue and followed up with protein expression. In addition to the cannabinoid receptors, the ECS also includes enzymes that *synthesize* AEA and 2-AG, namely, N-arachidonoyl phosphatidylethanolamine phospholipase D (NAPE-PLD) and diacylglycerol lipase (DAGL), respectively. It also includes enzymes that *degrade* AEA and 2-AG, such as fatty acid amide hydrolase (FAAH) and monoacylglycerol lipase (MAGL), respectively. Irrespective of exposure to drug or diet, there were no significant changes in mRNA transcript abundance for Cnr2 (CB2), Nape-pld, Faah and Mgll (Fig. 5a). With respect to cardiac Cnr1 (CB1) expression, there was an overall diet-effect reduction in cardiac Cnr1 mRNA (*p* = 0.0243; Fig. 5a). Consistent with these effects, immunoblots demonstrated a significant reduction (*p* = 0.0002) in cardiac CB1 protein expression in male offspring hearts exposed to the DHA + EPA diet (Fig. 5b). Specifically, offspring exposed to the DHA + EPA diet in conjunction with THC had significantly lower levels of cardiac CB1 protein expression relative to both VEH and THC offspring exposed to the control diet (*p* = 0.0026 and *p* = 0.0043, respectively). Since CB2 is the other canonical receptor and can share similar signalling pathways, we also evaluated CB2 protein levels via immunoblotting but did not observe any significant changes (Fig. 5b). For Daglα, there was a significant diet dependent decrease in Daglα mRNA levels (*p* = 0.0201). Associated with this, LC-MS data revealed that the maternal DHA + EPA diet significantly reduced cardiac 2-AG levels in offspring exposed to VEH (*p* = 0.0301) or THC (*p* = 0.0006), relative to the VEH control diet group (significant diet effect *p* = 0.0136; Fig. 5c). Moreover, offspring exposed to THC with the control diet also demonstrated a significant reduction (*p* = 0.0022) in cardiac 2-AG relative to the VEH control diet group (Fig. 5c). There were no changes in AEA concentrations between any groups statistically, but there was a notable p-value (*p* = 0.0632; Fig. 5c**)**.

## Discussion

The rate of cannabis use in pregnancy is rising and clinical studies suggest that this may lead to fetal growth deficits. Recent preclinical studies indicate that maternal exposure to THC alone leads to decreases in birthweight and subsequent reduction in cardiac function in male offspring^20,21^. In the present study, we provide evidence that a maternal diet supplemented with DHA and EPA can ameliorate both THC-induced fetal growth restriction and cardiac deficits in early postnatal life. Furthermore, we provide mechanistic insight into these cardioprotective effects of DHA + EPA and demonstrate that alterations in the endocannabinoid system may be intertwined with this cardioprotection.

Consistent with our previous models^19,20^, THC-exposed offspring (control diet) demonstrated a significant reduction in birthweight without significant alterations in gestational length, food intake, weight gain and litter size at birth^19,45,46^. There was a subtle drug-mediated decrease in food intake at the earlier time point, however, this is likely not a confounder given there were no alterations in maternal weight gain in between groups. Interestingly, pups exposed to the maternal DHA + EPA diet do not exhibit decreases in birth weight. This was not associated with increased gestational length as all time-pregnant rats gave birth on GD 22. These findings are consistent with clinical studies demonstrating that DHA + EPA can increase fetal growth due to enhanced biometric growth trajectories during gestation^33^. Previous clinical studies have also found that maternal consumption of essential fatty acids (e.g. DHA) can increase fetal weights^38^ and even reduce the rates of low birthweight (< 2500 g) babies^47^. However, the exact mechanism remains elusive. Aside from direct effects on the fetal growth, the protective effects of DHA and EPA could potentially be mediated by anti-inflammatory properties in the placenta. This has been reported in preclinical models^34^, which demonstrate that an omega-3 diet increases maternal and placental levels of DHA and EPA associated with a reduction in placental oxidative stress, culminating in improved in fetal and placental growth^34^. However, further studies on the placenta are needed to confirm this. With respect to the hearts at birth, unlike our previous study^20^, maternal THC exposure did not lead to a significant reduction in heart to body weight ratio. This may be attributed to a dietary differences in the current experimental model given that the current control diet is different from the chow provided (ProLab RMH 3000) from the animal facility in our previous study^20^. A new control diet for the current study was created to employ a more suitable control/comparator to a custom omega-3 diet. Our control diet currently has ~ 0.6% omega-3 content, while the previous Prolab RMH 3000 diet has 0.34%. This difference could confer to a greater baseline of protection in the control group of our current model, masking subtle changes.

The THC-exposed offspring also demonstrated complete catch-up up growth by PND21. We hypothesize catch-up growth acts as a second insult that potentiates the deficits in the heart, as previously demonstrated^20,48,49^. Indeed, our current study recapitulated the functional deficits (i.e. decreases in cardiac output and stroke volume) previously observed in PND21 offspring exposed to THC during gestation^20^. Interestingly, the perturbations in systolic function were not observed between groups in offspring exposed to the maternal DHA + EPA diet, and even significantly increased in certain parameters relative to the THC control diet group. This improvement is in agreement with previous preclinical models of cardiac insults (e.g. pressure overload, MI) demonstrating that DHA, EPA, or both^27,28,30,50–52^ can improve cardiac function post-insult. In the current study, it is noteworthy that these young animals do not exhibit overt heart failure/pathology at this timepoint. This is a known phenomenon in fetal growth-restricted neonates where they exhibit *subclinical* cardiac dysfunction due to intrauterine circulatory adaptations and/or direct insult by a drug/toxin^53,54^. Moreover, this is thought to be an early indicator of a compromised heart, potentially increasing the likelihood of developing cardiovascular disease later in life^53,54^.

Associated with the improved cardiac function, the maternal DHA + EPA dietary supplementation also led to marked increases in DHA and EPA along with a decrease in AA in the livers of PND21 offspring, most of which translated into the heart. Notably, EPA could not be quantified in our heart tissues at PND21, however, it is tempting to speculate this will follow a similar increase as observed in the liver. This speculation is supported by previous studies, which indicate that supplementation with DHA and EPA leads to significant increases in cardiac DHA and/or EPA in adult rodents^27,30^. Furthermore, similar to our observed outcomes, these studies also reported that omega-3 supplementation led to reductions in free-form AA along with its proinflammatory lipid mediators, which ultimately ameliorated inflammation and cardiac remodelling^27^. Unexpectedly, a reduction in cardiac AA was also observed in our offspring exposed to THC alone. This is surprising given AA is usually increased upon insult (e.g. pressure overload and MI)^27,30^. However, one important distinguishing factor regarding this particular prenatal insult is that THC can modulate the endocannabinoid system, which fundamentally relies on AA to produce endocannabinoids^55^. Studies in the heart are limited, but chronic THC exposure decreases the availability of free AA in the nervous system^55^. Of course, offspring in this current study are no longer exposed to THC in postnatal life but we have previously published that gestational cannabinoid exposure can lead to long-lasting alterations in the cardiac ECS (e.g. CB2 expression) in postnatal life^43^. In addition, the reduction in AA may also be attributed to augmented production of AA-derived pro-inflammatory lipid mediators. However, further studies quantifying these lipids are needed to confirm this.

The cardioprotective mechanistic underpinnings of DHA and EPA are vast, as reviewed in refs^56,57^. For example, DHA and EPA have been shown to downregulate expression of inflammatory genes through inhibition of NF-κB^58^ and PPARα/γ^59^ signalling as well as prevent NLRP3 inflammasome-dependent inflammation^60^. In addition, DHA and EPA produces pro-resolving lipid products (e.g. resolvins, protectins and maresins) that mediate inflammation while also lowering cardiac AA. A proposed mechanism of action for DHA and EPA is through competition/inhibition of the enzymatic production (i.e. cyclooxygenases) of AA-derived pro-inflammatory lipid mediators which results in preferential production of DHA and EPA-derived anti-inflammatory mediators^57^. Interestingly, we found that irrespective of drug treatment, the DHA + EPA diet significantly decreases gene expression of Cox-2. Previous studies have shown that DHA and EPA can inhibit Cox2 production of prostaglandins from AA^61^. Whether or not our animals exposed to the DHA and EPA diet had a reduction in pro-inflammatory mediators (*i.e*. prostaglandins) remains unknown. We also found that prenatal THC increases cardiac mRNA levels of Tnfα, but this was not ameliorated with our DHA + EPA diet. However, there was a significant diet dependant reduction in the expression of Ccl2 (MCP-1). This is of interest considering that omega-3 supplementation after a cardiac insult (i.e. pressure overload or MI) has been previously demonstrated to decrease cytokine (i.e. Ccl2^50^) gene expression and diminish inflammation, culminating in into reduced cardiac remodelling and improved cardiac function^27,50^.

Up to this point, we have demonstrated that maternal DHA and EPA ameliorated THC-induced decreases in cardiac function and enhanced postnatal fatty acid profiles that are associated with known anti-inflammatory outcomes in the heart. It is also known that these processes are entwined throughout cardiac remodelling. Indeed, we demonstrated that perinatal THC exposure significantly increased collagen content (COL-1 and COL-3) in PND21 male offspring hearts, which is in line with what we previously reported^20^. Intriguingly, this was significantly decreased when offspring were exposed to a maternal DHA + EPA-supplemented diet, which may underlie the improvement in systolic function observed. This outcome is consistent with the anti-fibrotic potential of DHA and/or EPA demonstrated in clinical^26^ and preclinical^27,62,63^ studies. In addition, we also observed diet-effect inhibition for protein expression of cardiac MMP-1. A previous study of hypoxia-induced FGR found increases in neonatal cardiac MMP-1^64^. MMPs may also increase in after an insult (i.e. MI), however, we did not observe any changes in MMP-1 in our growth-restricted offspring (e.g. THC-exposed offspring). Given that MMP-1 is a collagenase that breaks down COL-1 and COL-3, it is conceivable that our diet-induced reductions in MMP-1 are attributed to a lack of need to break down collagen. With respect to morphological cardiac remodelling, contrary to our previous findings, we did not observe subtle increases in left ventricular wall thickness^20^. As previously eluded to, this could likely be due to higher omega-3 content in our custom control diet group, potentially improving the subtle aberrations that would otherwise be observed with standard chow.

As mentioned earlier, both THC exposure as well as dietary DHA and EPA can impact the endocannabinoid system. Coincidentally, emerging evidence has drawn links with dietary n-3, the endocannabinoid system, and its role in cardioprotection^44^. While exposure to THC alone did not alter the gene expression of the endocannabinoid system, maternal DHA and EPA supplementation, particularly in combination with THC, led to a significant reduction in gene and protein expression of CB1. Similar effects have been observed on CB1 expression in epididymal fat^65^. Although the role of CB1 in cardiovascular pathologies is often conflicting, preclinical studies have found some benefits with inhibition. For example, blockade of the CB1 receptor has been shown to inhibit doxorubicin-induced cardiotoxicity^66^. Moreover, another study in rats found that rimonabant (selective CB1 antagonist) improved systolic function in hypertensive obese rats and also reduced collagen content and improved cardiac function in rats post-MI^67^. However, further studies are required to examine these effects using the current model. Interestingly, we demonstrated that the DHA and EPA diet significantly decreased cardiac 2-AG. Previous studies have demonstrated that krill oil (rich in DHA and EPA) significantly decreases AEA and 2-AG in heart and adipose tissue in a dose-dependent manner, ultimately ameliorating metabolic disturbances^68^. Studies suggest that reductions in AEA and 2-AG are associated with reduced AA, with no changes in ECS-degrading enzymes^69^. This is consistent with the fact that 2-AG is reliant on AA for synthesis. Indeed, the diet-induced significant reduction in cardiac AA observed in offspring directly mirrors the reduction in 2-AG. Moreover, an interesting finding that warrants further discussion is that the offspring exposed to THC alone demonstrate reduction in AA as well as 2-AG, similar to the DHA + EPA diet groups. While both the offspring exposed to THC alone and groups exposed to DHA + EPA exhibit reductions in 2-AG (a full agonist at CB1 and CB2), the DHA + EPA group may have a further dampened ECS response at CB1 and possibly greater activity at the cardioprotective CB2, potentially lending itself to greater protection. Notably, given that 2-AG has cardioprotective potential^70,71^, the reduction in 2-AG could on one end, be indicative of a diminished protective response in THC-exposed offspring and, on the other end, be indicative of a lack of need for the cardioprotective response in our DHA + EPA groups.

Our study has a few limitations, firstly, we did not assess female rats therefore we could not determine whether females are impacted and/or benefit from the DHA + EPA diet. We focused on males in this study since our previous study reported cardiac aberrations in males^20^. Notably, previous work from our group demonstrated that female offspring exposed to THC in gestation do not exhibit differences in circulating estrogen and testosterone compared to control, but it remains plausible that there might be some underlying epigenetic differences at this early age^72^. Another limitation is that we did not measure E and A waves via echocardiography and therefore could not evaluate diastolic function.

Future studies should assess whether shorter windows of intervention (i.e. during lactation only) also leads to cardioprotection. This will address the question of whether our diet is beneficial after *in utero* THC exposure. In addition, future studies should determine if these benefits persist into adulthood. Additional studies could also delineate the definitive role of inflammation in the context of maternal THC and omega-3 exposure. Examining DHA, EPA, AA metabolites and lipid mediators (e.g. PD1/2, RvD1/2, 18-HEPE, PGE_2_) as well as macrophage polarization in offspring hearts could be informative.

In conclusion, our data demonstrates that gestational THC exposure leads to reduced cardiac function, increased TNFα gene expression and collagen protein expression in male offspring PND21 hearts. We provide, early preclinical evidence that maternal dietary omega-3 supplementation prevents THC-induced deficits in both fetal growth and postnatal cardiac function. Furthermore, we provide mechanistic insight from our DHA/EPA diet specifically relating to (1) changes in fatty acid composition in the liver and heart, (2) the potential to suppress inflammatory response in the heart, (3) reduced markers of cardiac remodeling and (4) alterations in the endocannabinoid system in the heart. In the larger context, this intervention raises new questions from a harm reduction standpoint. However, we want to emphasize that given the current evidence, cessation of cannabis is still the best way to reduce the potential risks of adverse effects on the fetus. It is also important to reiterate that this study reports the beneficial *cardiac outcome*s of a DHA + EPA-enriched diet and does not investigate whether the omega-3-enriched diet will ameliorate other known negative implications of perinatal THC exposure (e.g. neuropsychiatric and/or metabolic perturbations). Nonetheless our study proposes the exciting possibility that DHA and EPA in perinatal/postnatal life may be efficacious in ameliorating the postnatal deficits in the heart of offspring that were exposed to cannabinoids in utero.

## Methods

### Experimental animal model

All animal procedures were conducted in accordance with the guidelines and standards of the Canadian Council on Animal Care. Animal Use Protocol (AUP No. 2023 − 129) was approved and monitored by the Western University Animal Care Committee. All investigators understood and followed the ethical principles outlined by Grundy^73^ and the methods were performed in accordance with Animal Research: Reporting of In Vivo Experiments (ARRIVE) guidelines^74^. Time-pregnant Wistar rat dams were purchased from Charles River (La Salle) and were maintained at 22 °C on a light/dark cycle (12:12 light: dark) with access to food and water ad libitum. Pregnant rats arrived at the animal facility on gestational day (GD) 3 and were left to acclimatize for 3 days. Throughout gestation, maternal food intake and body weight were monitored to study maternal weight gain and nutrition, as previously published^19,43^. The length of gestation was also measured to account for pre-term labor.

At birth, litters were counted and culled to 8 for uniform postnatal nutrition. Of the culled pups, hearts and total body were weighed. At postnatal day (PND) 21, male offspring underwent echocardiography to assess cardiac function. At this timepoint, males were also euthanized with an overdose of pentobarbital i.p. (100 mg/kg) followed by decapitation. Left ventricular heart tissue was dissected and flash-frozen in liquid nitrogen for further mRNA, protein and fatty acid analysis. We focused on males in this study since our previous study reported cardiac aberrations in males^20^.

### Drug and diet preparation

Δ9-THC was received in ethanol, which was evaporated with nitrogen gas. From there, THC was mixed with cremaphor and saline in a 1:1:18 ratio (THC: cremaphor: saline) to the experimental concentrations of 3 mg/kg along with vehicle saline (1:18, cremaphor: saline). Similar to our previously published prenatal THC exposure models^19,20,72,75–77^, pregnant dams were randomly assigned to a treatment group and administered either 3 mg/kg THC or vehicle control daily via i.p. injection from GD6 to GD22 (birth). Concurrently, as separate groups, we also gave these pregnant rats either a control diet or a DHA + EPA-supplemented diet from the time of arrival (GD3) until pups were weaned at postnatal day (PND) 21.

This diet intervention timeline was selected to ensure coverage throughout the entire course of gestational THC exposure. This diet also encompasses the first 21 days of postnatal life as we have previously reported that this is when signs of cardiac deficits begin to manifest^20^. The caloric differences between the intervention diet and the control diet are negligible. The combined gram weight of DHA and EPA (70% DHA and 30% EPA, algae-derived, Algarithm Inc.) is ~ 1.2 g per 1 kg of chow, and the animals consume about 25–30 g of chow per day, thus the additional daily caloric intake is most likely less than half a calorie in the intervention diet. Again, animals given the intervention diet were supplemented with ~ 1.2 g/kg of DHA and EPA in their chow, which equates to approximately 1.7% of total fats. Similarly, a study using 1.46% DHA and EPA (out of total fats) content demonstrated reduced cardiometabolic risk in male rats^78^. Also, both the control diet and DHA + EPA-enriched diet have a similar omega-3:omega-6 ratio up to 1:5 and 1:4, respectively. The similarity between these two ratios is to ensure that the control diet animals are not simply being deprived of essential omega-3s. In addition, it has been previously shown a ratio of 1:5 is at the cusp of the ideal ratio for cardioprotection^79,80^. See Supplementary Table S1 for the diet composition.

The window of THC exposure was selected because it has been demonstrated that THC exposure before GD5.5 adversely impacts implantation in rodents^81^. Moreover, this dose was selected as similar doses (2.5 mg/kg i.p.) in female rats leads to a plasma concentration (~ 25 ng/mL, 1 h post-injection) comparable to the that of human cannabis smokers (5.2–28 ng/mL 1 h post-inhalation with 6.8% Δ9-THC)^82,83^.

### Echocardiography

The Vevo2100 Ultrasound Imaging System was utilized to measure cardiac function. A two-dimensional echocardiographic footage was captured blinded in PND21 offspring. Parasternal short axial and long axial views were captured using a 40 MHz linear transducer, as previously published^20,43^. During echocardiography, animals were sedated using isoflurane (0.5–1.5%) through a nose cone. Heart rate was measured using electrode probes on the extremities and body temperature was maintained with a heating dock and monitored using a rectal probe. Real-time images obtained in the short axial view were used to measure parameters such as left ventricular posterior wall (LVPW) and anterior wall (LVAW) thickness as well as left ventricular interior diameter (LVID) at systole and diastole. Estimates were made for cardiac function parameters (e.g., stroke volume, ejection fraction and cardiac output) using left ventricular traces in systole and diastole.

### Fatty acid extraction and high-performance liquid chromatography mass spectrometry (HPLC-MS)

Wistar 3-week rat heart ventricles were collected, flash frozen in liquid nitrogen, and stored at -80 °C until sample processing. The extraction and HPLC-MS analysis was adapted from Reinicke et al.^84^. Briefly, 50–70 mg of liver or heart tissue was weighed. Then homogenization in hexane and isopropyl alcohol was performed with sonication. Samples were extracted in 650 µL of 60% hexane, 40% isopropyl alcohol, 0.1% formic acid. Samples were placed on mixer rotator and then centrifuged. The supernatant was transferred, and solvent was evaporated in gentle stream of N_2_ gas. Evaporated samples were stored in a -20 °C freezer prior to LC-MS analysis. Samples were reconstituted in mobile phase A for LC-MS analysis.

Samples were injected as prepared in the extraction method and separated using an Agilent 1100 high-performance liquid chromatography (HPLC) system comprising a degasser, quaternary pump, autosampler, and column heater (Santa Clara, CA, U.S.A.) with C18 reverse phase column (bioZen 2.6 μm particle size Peptide XB-C18 (150 × 4.6 mm) (Phenomenex, U.S.A.). Mobile phase was composed of two solutions: (A) 63% LC-MS grade water, 37% ACN, and 0.02% formic acid and (B) contained 50% ACN, 50% isopropanol. The gradient was set as follows: 20–90% of eluent B over 8 min, 90% eluent B for 2 min, 100% eluent B for 2 min and reequilibration of the column. The flow rate was set to 0.5 mL/min and the temperature of the column was kept at 35 °C. Mass spectrometry was performed with a Waters Micromass QTOF Ultima Global mass spectrometer (Waters, Milford, MA, U.S.A.) operated in negative ion mode using the ESI source. The survey scan was acquired from 200 to 400 Da in time-of-flight (TOF) MS mode over a 20-minute run time. DHA, AA, and EPA standards were used to confirm the suitability of the LC- MS system. Tandem mass spectrometry was performed on analytical standards and compared to the MS/MS in the control heart and liver sample for identification. DHA and AA were identified in both the liver and heart, whereas EPA was identified only in the liver.

For LC- MS experiments, each biological replicate (*n* = 4–5) was analyzed in triplicate to produce three technical replicates. Extracted mass chromatograms were generated from the LC-MS data for each of the eluted masses for each sample and compared to an external calibration curve. The spectra were analyzed by Skyline^85^ with TIC normalization.

### Endocannabinoid extraction and HPLC-MS

Wistar 3-week rat heart ventricles were collected, flash frozen in liquid nitrogen, and stored at -80 °C until sample processing. Homogenized rat livers and heart ventricles (~ 0.1 g) were placed into a 2.0 mL microcentrifuge tube and spiked with the mass-labeled internal standard d4-2-AE (20 µL of 7.5 ng/µL). Extractions were performed by adding 1.35 mL of 35:65 H_2_O: ACN to each tube, sonicating in an ice bath for 12 min, vortexing for 1 min and centrifuging at 14,800 rpm for 15 min. The supernatant was removed, and the remaining tissue was re-extracted as before. Extracts were combined and reduced in volume to 1.5 mL with a gentle stream of ultra-high purity N_2_ prior to HPLC-MS/MS analysis.

The detection and quantitation of target analyses were based on a modified version of a previously published validated method^86,87^. In brief, for the endocannabinoids, separations were achieved using an XSelect^®^ HSS T3 C18 column (2.5 μm, 2.1 mm x 50 mm) in conjunction with a VanGuard^®^ HSS T3 C18 cartridge (3 mm, 2.1 mm x 5 mm) and ionization in the positive electrospray mode. The HPLC used was an Agilent 1100 (Palo Alto, CA) with a CTC PAL autosampler. The HPLC was coupled to a Sciex 365 triple quadrupole mass spectrometer retrofitted with an HSID Ionics EP + orthogonal ionization source. The following multiple reaction monitoring ion transitions were monitored: 2-AG: m/z 379.3, m/z 287.2; 2-AE: m/z 348, m/z 287.2 and d4-2-AE: m/z 352.3, m/z 287.2. Method detection limit for AEA and 2-AG, determined according to the procedure described in the Euachem Guide for Method Validation^88^ were 9.2 and 63.8 ng/g, respectively.

### RNA extraction and qPCR

Total RNA was isolated from rat heart tissue homogenization using TRIzol (Invitrogen) and chloroform (Sigma-Aldrich) extraction. RNA was diluted to 2 µg for reverse-transcriptase with a High-Capacity cDNA (complementary DNA) Reverse Transcriptase Kit (No. 4368814; Applied Biosystems) to make cDNA. Primer sets were designed using the NCBI Primer-BLAST tool (see **Supplementary Table S2** for primer sets). SsoFast Eva green supermix (Bio-Rad) and Bio-Rad CFX384 Real-Time System were used with cyclic conditions set at 95 °C for 3 min, followed by 43 cycles of 95 °C for 15 s, 58 °C for 30 s, and 72 °C for 30 s. All primer sets used were tested for one distinct melt peak and determined to fall within 90–110% primer efficiency. Relative transcript (mRNA) abundance obtained for all target genes of interest was normalized to the geometric means of Gapdh and β-actin. The ΔCt values for each target were calibrated to the average of the control. Relative gene expression was then calculated for each primer set as determined by the formula 2^−ΔΔCt^, where ΔΔCt was the normalized value.

### Protein extraction and immunoblots

Total protein from rat heart tissue was extracted by homogenization and sonication in RIPA buffer solution (50 mM Tris-HCl, pH 7.4, 150 mM NaCl, 1 mM EDTA, 1% Nonidet P40, and 0.25% C_24_H_39_NaO_4_) with protease and phosphatase inhibitors (40 mM Na_3_VO_4_, 40 mM Na-pyrophosphate 20 mM NaF, and 200 mM β-glycerophosphate disodium salt hydrate). Total protein samples were quantified using a BCA protein assay kit (No. 23225; Thermo Scientific) and diluted to 20 µg/well (well = 10 µL of mix) of protein. A pooled sample was prepared with contributions from every sample across treatment groups. Protein samples were separated via gel electrophoresis using 4–12% (w/v) Bis-Tris gradient gels (Invitrogen) and transferred onto polyvinylidene difluoride membranes at 100 V for 2 h. Ponceau S (0.1%) staining was applied to membranes and imaged to ensure equal loading and proper transfer of proteins. Membranes were blocked with 5% non-fat milk for 1 h at room temperature and then probed overnight at 4 °C with a primary antibody diluted in blocking agent. See **Supplementary Table S3** for the full antibody list with dilutions. On the next day, HRP-linked secondary antibody (**Supplementary Table S3**) was diluted in blocking agent and rotated at room temperature for 1 h. Immunoreactive bands were detected using the Super Signal West Dura Chemiluminescent Substrate (Thermo Fisher Scientific) and then imaged on Chemi Doc Imager (Bio-Rad). For quantification, relative band density was normalized to total protein using Ponceau S staining and quantified using the Image Lab software 6.0.1 (Bio-Rad). When two blots were used for the analysis of a target/antibody, a pooled sample ratio between the two blots was used to normalize densitometry values between blots.

### Quantification and statistical analysis

For body weights at PND1, each data point represents an average of 3–4 pups from 1 mom, as such data is represented as means ± standard error of the mean. All other experiments involving maternal, echocardiographic, LC-MS, qPCR, immunoblotting data are represented as mean ± standard deviation. In addition, in experiments involving echocardiographic, LC-MS, qPCR, immunoblotting data, the number of pups/offspring hearts/livers utilized correspond to the number of dams. As such, each data point from an offspring corresponds to a single and different dam. This was performed to avoid litter bias. To analyze the effects and interaction of maternal THC exposure and the DHA + EPA diet, a two-way analysis of variance (ANOVA) with post-hoc Tukey’s was used. The alpha value used was 0.05. All statistical tests were completed with GraphPad Prism 9 version 9.3.1. Specific statistical details regarding tests used and exact sample sizes can be found in the figure legends.

## Electronic supplementary material

Below is the link to the electronic supplementary material.

Supplementary Material 1

## Data Availability

The key datasets generated during and/or analysed during the current study are available in the supplementary information file. Further data may be obtained from the corresponding author on reasonable request.
