# Supplementary material for: Maternal dietary DHA and EPA supplementation ameliorates adverse cardiac outcomes in THC-exposed rat offspring
Source: Sci Rep. 2025 Mar 10;15:8316. doi: 10.1038/s41598-025-92844-6 (PMC11894106; doi:10.1038/s41598-025-92844-6)
Supplement: Supplementary file 1 — Supplementary Material 1 [file 41598_2025_92844_MOESM1_ESM.docx]

**Maternal dietary DHA and EPA supplementation ameliorates the adverse cardiac outcomes in rat offspring exposed to THC in gestation**

Kendrick Lee, Mohammed H. Sarikahya, Samantha L. Cousineau, Ken K.-C. Yeung, Amica Lucas, Kara Loudon, Thane Tomy, Gregg T. Tomy, David R.C. Natale, Steven R. Laviolette and Daniel B. Hardy

**Supplementary Information**

**Supplementary Table S1**. Composition of the Control and Omega-3 Diets

| Class description | Ingredients |  | Control Diet | Omega-3 Diet |
| --- | --- | --- | --- | --- |
| Protein | Casein, Lactic, 30 Mesh |  | 200.00 g | 200.00 g |
| Protein | Cystine, L |  | 3.00 g | 3.00 g |
| Carbohydrate | Starch, Corn |  | 381.00 g | 381.00 g |
| Carbohydrate | Dextrose, Monohydrate |  | 150.00 g | 150.00 g |
| Carbohydrate | Lodex 10 |  | 110.00 g | 110.00 g |
| Carbohydrate | Sucrose, Fine Granulated |  | 4.00 g | 4.00 g |
| Fiber | Solka Floc, FCC200 |  | 75.00 g | 75.00 g |
| Fiber | Raftiline HP |  | 25.00 g | 25.00 g |
| Fat | Soybean Oil, USP |  |  |  |
|  | Composition: | 51% linoleic acid | 35.7g | 35.7g |
|  |  | 7-10% α-linolenic acid | 4.9-7g | 4.9-7g |
|  |  | 23% oleic acid | 16.1g | 16.1g |
|  |  | 10% palmitic acid | 7g | 7g |
|  |  | 4% stearic acid | 2.8g | 2.8g |
| Fat | Algae, Algarithm Inc. |  |  |  |
|  | Composition: | 70% Docosahexaenoic acid | 0.00 g | 0.906 g |
|  |  | 30% Eicosopentanoic Acid | 0.00 g | 0.282 g |
| Mineral | [S10026B](https://researchdiets.com/formulas/S10026B) |  | 50.00 g | 50.00 g |
| Vitamin | Choline Bitartrate |  | 2.00 g | 2.00 g |
| Vitamin | [V10001C](https://researchdiets.com/formulas/V10001C) |  | 1.00 g | 1.00 g |
| Dye | Dye, Blue FD&C #1, Alum. Lake 35-42% |  | 0.03 g | 0.00 g |
| Dye | Dye, Yellow FD&C #5, Alum. Lake 35-42% |  | 0.03 g | 0.00 g |
| Dye | Yellow FD&C #5, Alum. Lake 35-42% |  | 0.00 g | 0.04 g |
| Dye | Red FD&C #40, Alum. Lake 35-42% |  | 0.00 g | 0.01 g |
| Protein | Casein, Lactic, 30 Mesh |  | 200.00 g | 200.00 g |
| Protein | Cystine, L |  | 3.00 g | 3.00 g |

**Supplementary Table S2**. Forward and reverse rat primer sequences for real-time qPCR

| **Gene** | **Forward**  **(5’ to 3’)** | **Reverse**  **(5’ to 3’)** | **GenBank** |
| --- | --- | --- | --- |
| Ptgs1 (Cox-1) | CTTAGGCCATGGGGTAGACCT | GACGCCTGTTCTACGGAAGG | NM_017043.4 |
| Ptgs2 (Cox-2) | ACGTGTTGACGTCCAGATCA | GGCCCTGGTGTAGTAGGAGA | NM_017232.4 |
| Alox15 (12/15 Lox) | CTGTCGGGACTCGGAAGCA | GCCCTGAACCCATCGGTAAC | NM_031010.2 |
| Tnfα | ATGGGCTCCCTCTCATCAGT | GCTTGGTGGTTTGCTACGAC | NM_012675.3 |
| Ccl2 | CCCAGAAACCAGCCAACTCT | GGGCATTAACTGCATCTGGC | NM_031530.1 |
| Ccl3 | CATATGGAGCTGACACCCCG | CTCTTGGTCAGGAAAATGACACC | NM_013025.2 |
| Il-6 | TCATTCTGTCTCGAGCCCAC | TGGCTGGAAGTCTCTTGCG | NM_012589.2 |
| Il-1b | TAGCAGCTTTCGACAGTGAGG | CTCCACGGGCAAGACATAGG | NM_031512.2 |
| Cnr1 (CB1) | CATTTCAAGCAAGGAGCACCC | GGTCTGTGGTGATGGTACGG | NM_012784.5 |
| Cnr2 (CB2) | AGGTTGCATTCCCAACAGAC | TTAGTTCCTCTGGGCAATGG | NM_001164143.3 |
| Faah | GTTCACCTTGGACCCTACCG | AGAAGGGAATCAGCGTGTGG | NM_001369126.1 |
| Napepld | AGCTTATGAGCCAAGGTGGT | AGCTAAGGCAAAAGTCCCCC | NM_199381.2 |
| Mgll (MGL) | ACCAACCCACTTTTCTGGCA | CAACCTCCGACTTGTTCCGA | NM_001398597.1 |
| Daglα | CAGTACGTGCTCTACGTGCG | AGACGACCATCCCGAGAGT | NM_001005886.2 |
| Beta actin | CGCGAGTACAACCTTCTTGC | CGCAGCGATATCGTCATCCA | NM_031144.3 |
| Gapdh | CTCTCTGCTCCTCCCTGTTC | CGATACGGCCAAATCCGTTC | NM_017008.4 |

**Supplementary Table S3**. Primary and Secondary Antibodies for Immunoblots

| Antibody | Source | Dilution | Catalog No. and Company |
| --- | --- | --- | --- |
| Collagen Type I (COL-1A1) | Rabbit Recombinant Monoclonal | 1:2000 | AB260043; Abcam |
| Collagen Type III (COL-3) | Rabbit Polyclonal | 1:500 | 22734-1-AP; Proteintech |
| MMP1 | Rabbit Polyclonal | 1:2000 | 103712AP; Proteintech |
| MMP2 | Rabbit Monoclonal | 1:1000 | D204T; Cell Signalling Technology |
| Cannabinoid Receptor 1 (CB1) | Rabbit Recombinant Monoclonal | 1:2000 | ab259323; Abcam |
| Cannabinoid Receptor 2 (CB2) | Rabbit Polyclonal | 1:2000 | ab3561; Abcam |
| Anti-rabbit secondary IgG HRP-linked (H + L chain) | Goat | 1:10,000 | 7074P2; Cell Signaling Technology |
| Anti-mouse secondary IgG HRP-linked (H + L chain) | Horse | 1:10,000 | 7076S; Cell Signaling Technology |

**
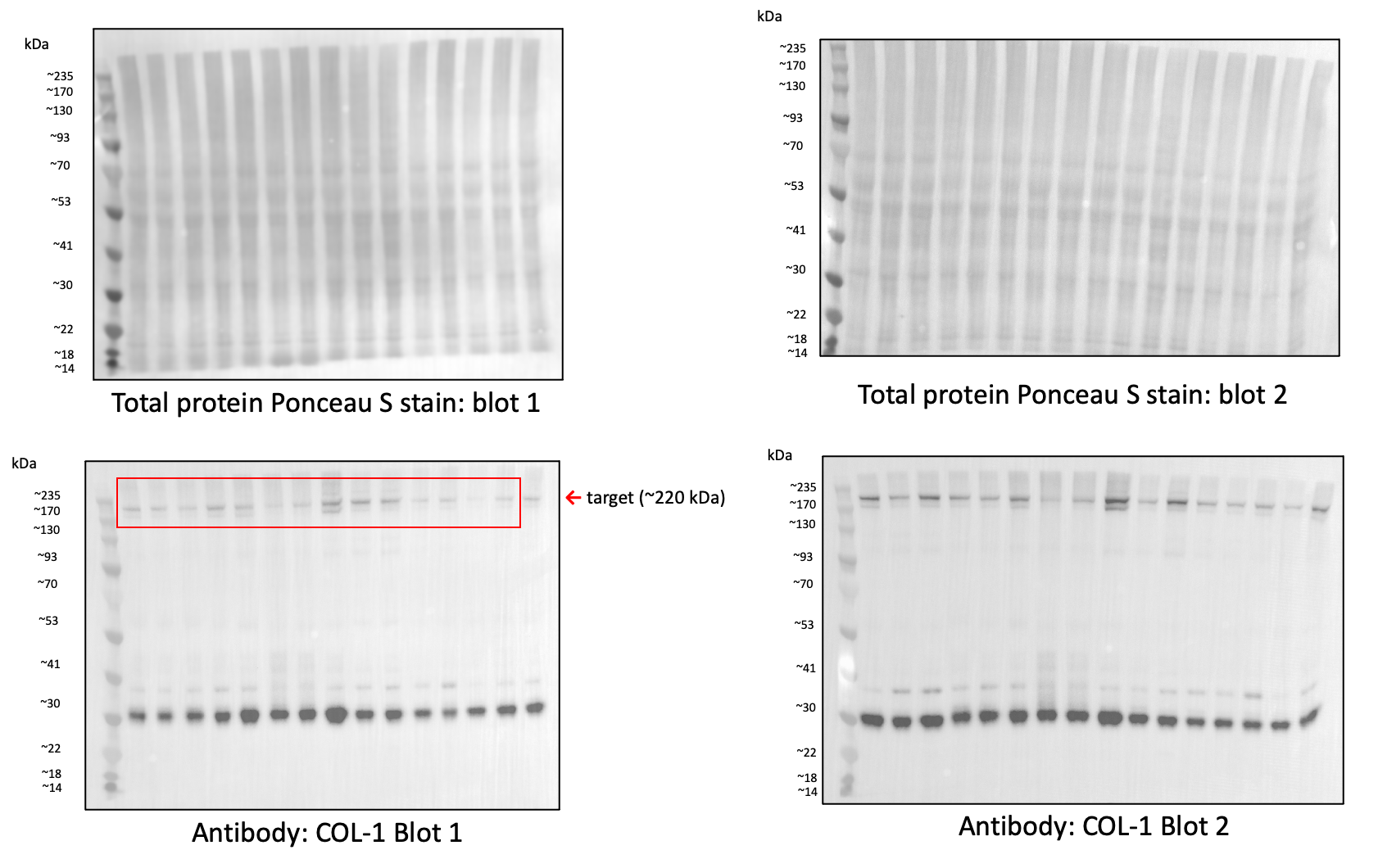
**

**Supplementary Figure S1.** Full immunoblots for COL-1 in male PND21 hearts. Red boxes indicate cropped portions of each immunoblot displayed in the manuscript figures. All targets were normalized to total protein Ponceau S staining. Last lane (far right) of each immunoblot was loaded with a pooled sample to normalize for semi-quantitative comparison between blots.

**
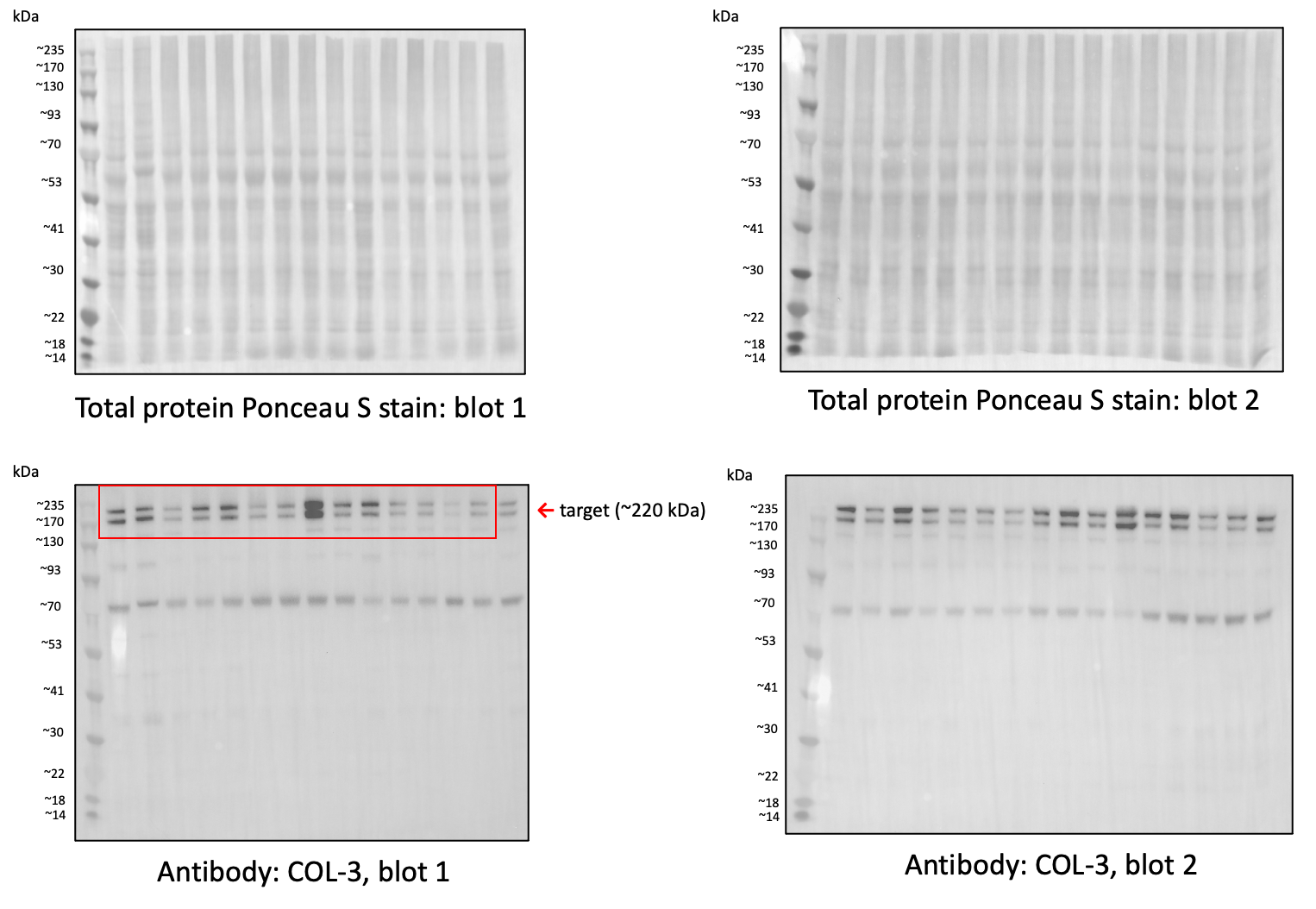
**

**Supplementary Figure S2.** Full immunoblots for COL-3 in male PND21 hearts. Red boxes indicate cropped portions of each immunoblot displayed in the manuscript figures. All targets were normalized to total protein Ponceau S staining. Last lane (far right) of each immunoblot was loaded with a pooled sample to normalize for semi-quantitative comparison between blots.

**
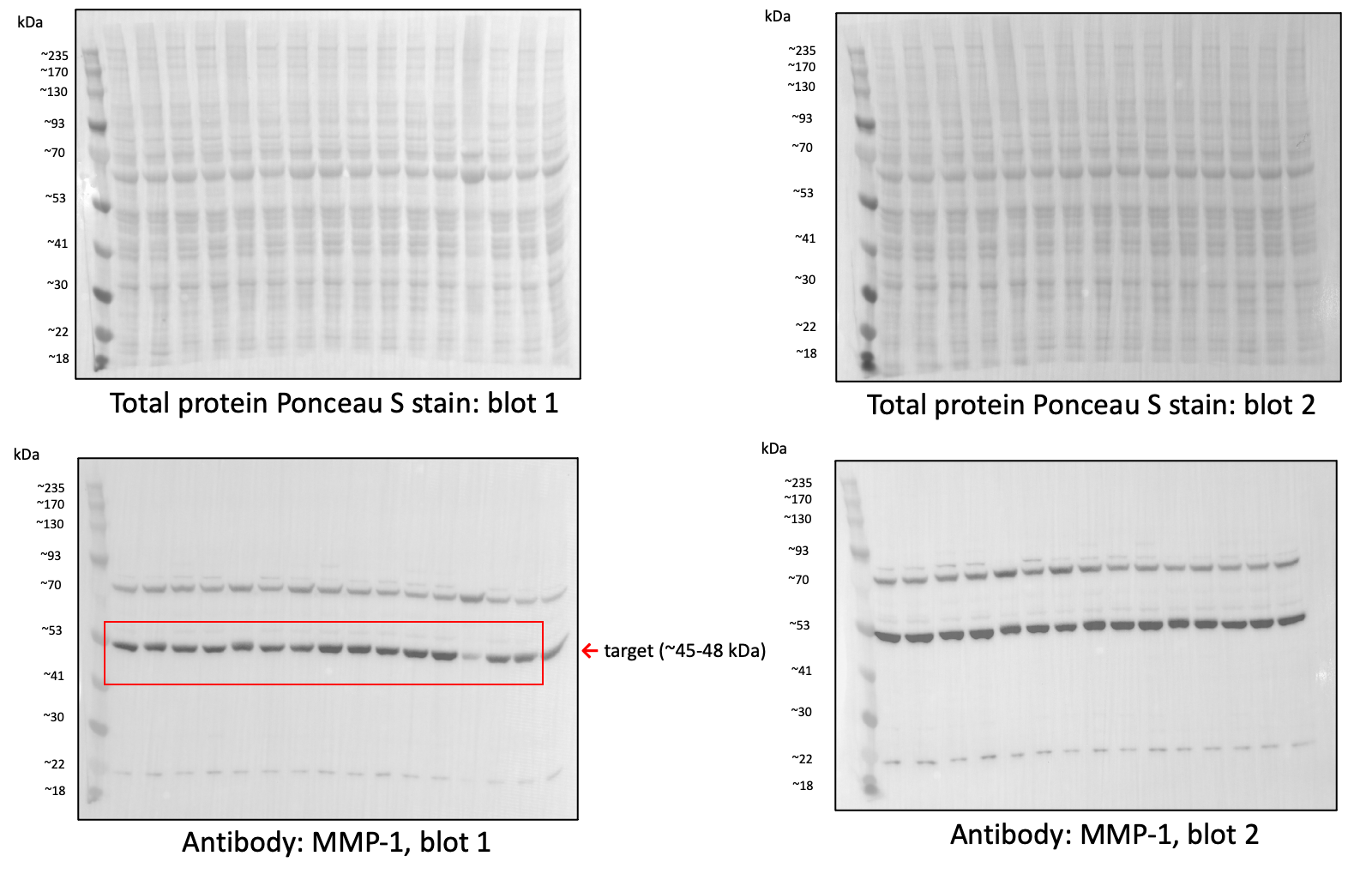
**

**Supplementary Figure S3.** Full immunoblots for MMP-1 in male PND21 hearts. Red boxes indicate cropped portions of each immunoblot displayed in the manuscript figures. All targets were normalized to total protein Ponceau S staining. Last lane (far right) of each immunoblot was loaded with a pooled sample to normalize for semi-quantitative comparison between blots.

**
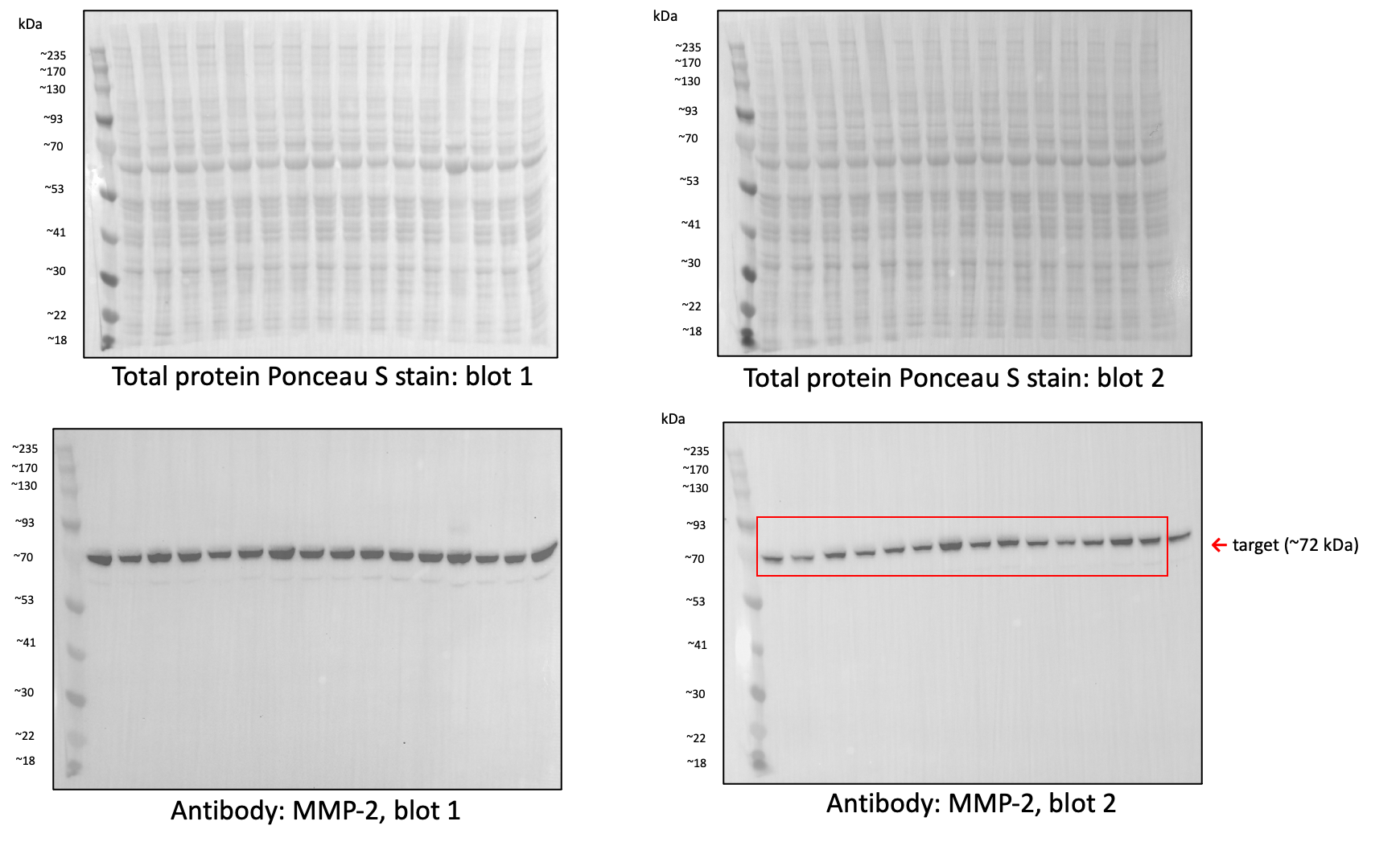
**

**Supplementary Figure S4.** Full immunoblots for MMP-2 in male PND21 hearts. Red boxes indicate cropped portions of each immunoblot displayed in the manuscript figures. Blots from MMP-1 were stripped and re-probed with MMP-2 primary antibody, hence targets were normalized to total protein Ponceau S staining from the previous blot (**Supplementary Figure S5)**. Last lane (far right) of each immunoblot was loaded with a pooled sample to normalize for semi-quantitative comparison between blots.

**
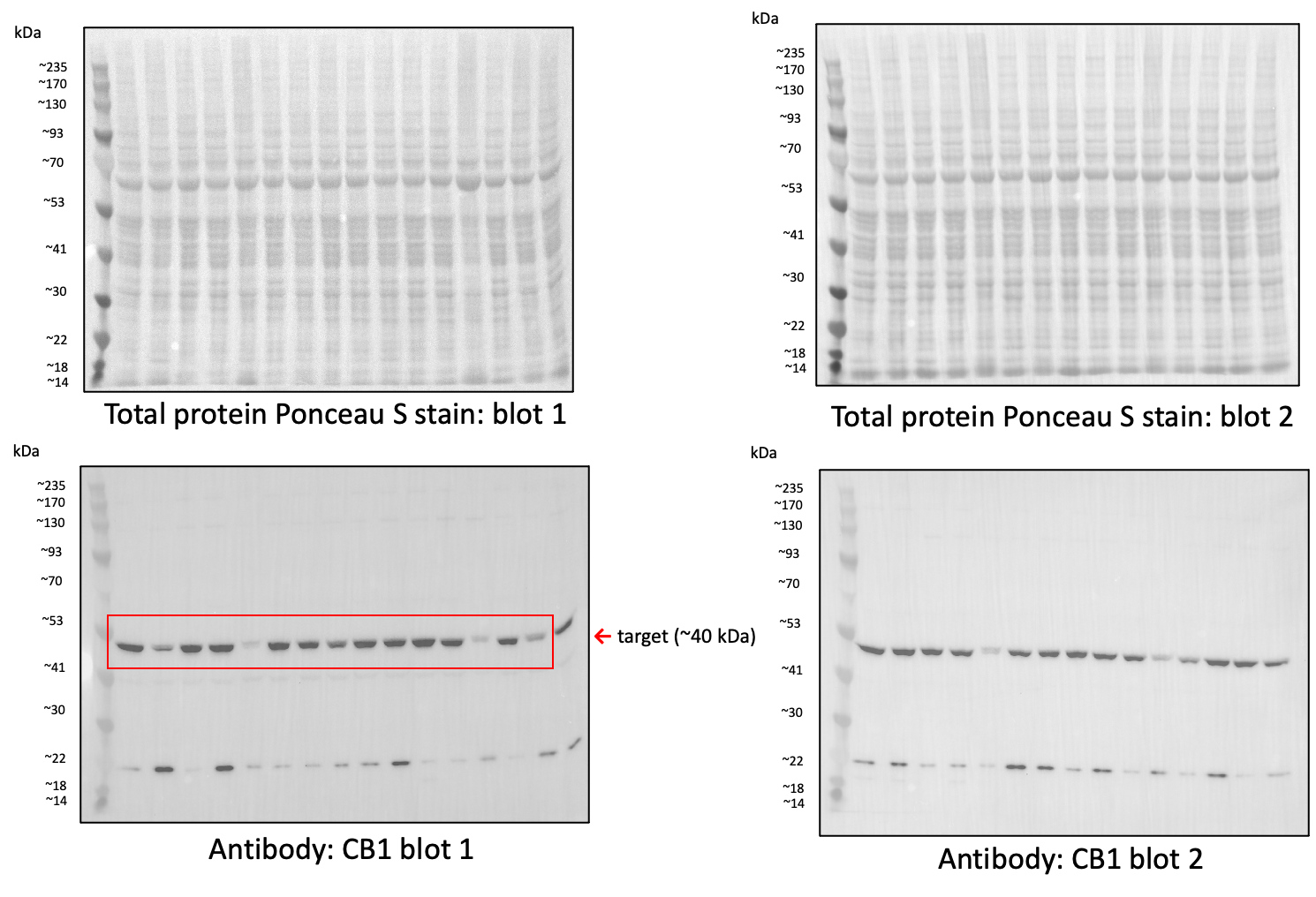
**

**Supplementary Figure S5.** Full immunoblots for CB1 in male PND21 hearts. Red boxes indicate cropped portions of each immunoblot displayed in the manuscript figures. All targets were normalized to total protein Ponceau S staining. Last lane (far right) of each immunoblot was loaded with a pooled sample to normalize for semi-quantitative comparison between blots.


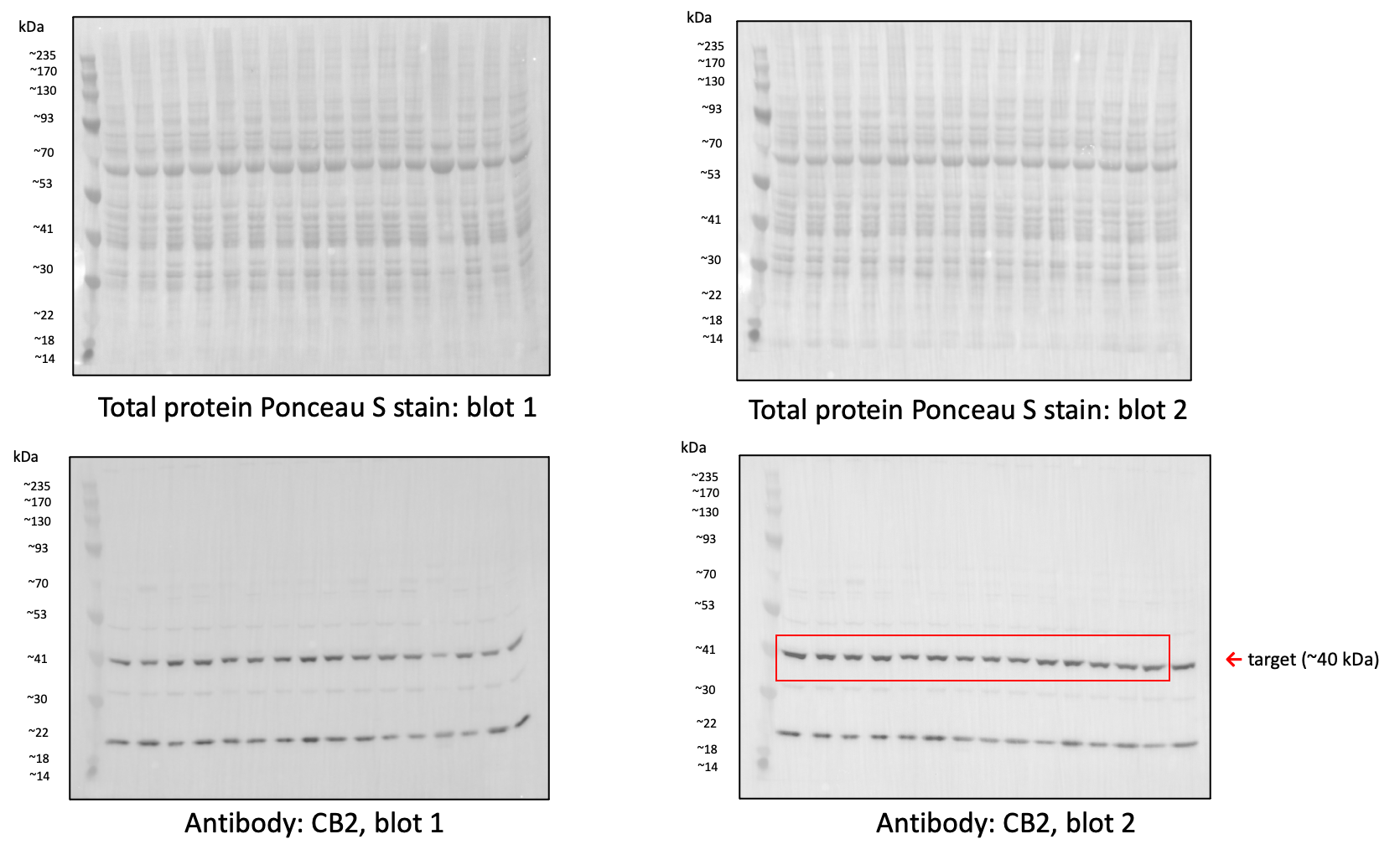


**Supplementary Figure S6.** Full immunoblots for CB2 in male PND21 hearts. Red boxes indicate cropped portions of each immunoblot displayed in the manuscript figures. All targets were normalized to total protein Ponceau S staining. Last lane (far right) of each immunoblot was loaded with a pooled sample to normalize for semi-quantitative comparison between blots.
